# Next steps in the development of a sibling adjustment scale: reply to Supianto

**DOI:** 10.1080/20473869.2025.2456803

**Published:** 2025-02-20

**Authors:** Linda K. M. Veerman, Stian Orm, Krister W. Fjermestad, Paula S. Sterkenburg, Agnes M. Willemen

**Affiliations:** a Clinical Child & Family Studies, Amsterdam Public Health, LEARN!, Vrije Universiteit Amsterdam, Amsterdam, The Netherlands; b Department of Psychology, Inland Norway University of Applied Sciences, Lillehammer, Norway; c Department of Psychology, University of Oslo, Oslo, Norway; d Frambu Resource Center for Rare Disorders, Siggerud, Norway; e Bartiméus, Doorn, The Netherlands

We thank the author for their Letter (Supianto, 2025) in response to our paper on the psychometric properties of the Negative Adjustment Scale (NAS) in siblings of children with chronic conditions (Veerman et al. 2024). The author thoroughly highlighted and elaborated on the critical points and future directions discussed in our paper. The Letter provides a clear overview of the NAS’s limitations and reinforces our conclusions. In particular, the author underscored the conceptual problems of the NAS and the absence of positive items. This stresses the need for developing a new scale that fits in with the current systemic paradigm emphasizing both positive and negative aspects of having a sibling with a disability (Hayden & Hastings, 2022). The underlined methodological problems call for a more robust measure that is usable across a broad range of cultures, age groups, and disabilities. This encouraged us to initiate developing a new sibling-tailored scale. Herein, we describe a guideline-based structured developmental process for this (Boateng et al. 2018).

It is essential to form an international team of sibling researchers. Involving clinicians, siblings, and parents is crucial in creating a measure that matches their needs and perceptions, that can also be used in clinical practice. Including representatives beyond Europe and North America and from ethnic minority groups is essential, because current scales are ethnocentric, as highlighted by (Supianto, 2025). This team should then develop the scale in three phases (Boateng et al. 2018) described below and summarized in Table 1.

**Table 1.** Overview of phases and steps in developing a new sibling-tailored measure.

Phases	Steps	Comments
Phase 0	Form a team of researchers, clinicians, siblings and parents	International team with diverse representation of cultures, ethnicities, and clinical settings.
Phase 1	1.1 Conceptualize the domain and dimensions	Based on experiences, theory and previous research in the sibling or broader field; include positive and negative elements and a systemic view.
	1.2 Determine the target group and versions	Consider age range and broad or specific disabilities; possible need for different versions for subgroups. Determine differences in informants’ versions.
	1.3 Identify relevant existing measures	Both possibly useful sibling-tailored and general measures.
	1.4 Select appropriate items from existing measures	Focus groups to select items. Translate items where needed.
	1.4 Generate new items based on previous studies	Focus groups to generate items in multiple languages based on previous qualitative studies and own experiences.
	1.5 Assess content validity	Evaluations by experts, siblings and parents.
Phase 2	2.1 Conduct cognitive interviews	Multiple rounds of interviews, adaptations in-between rounds.
	2.2 Administer items in a group of users at two datapoints	Include a diverse group of siblings and parents and a sufficient sample size.
	2.3 Determine basic psychometric properties	Inter-item correlations, item-total correlations, exploratory factor analysis.
	2.4 Omit and adapt items	Based on findings in Phase 2.1–2.3.
Phase 3	3.1 Assess dimensionality	Confirmatory factor analysis, invariance analysis.
	3.2 Assess reliability	Internal consistency and test-retest reliability.
	3.3 Assess validity	Criterion and construct validity.

Note. Based on Boateng et al. 2018.

In the first phase, the project team starts with conceptualizing the domains and dimensions that the proposed scale should measure, possibly with a Delphi method. In Veerman et al. (2024) we built on the conceptualization of other researchers when defining sibling adjustment not as an outcome, but as the process of how a sibling appraises and adapts to the situation of having a sibling with a disability. However, in a further effort to conceptualize sibling adjustment, other related concepts might be considered as well, such as ‘sibling quality of life’ (e.g. Moyson & Roeyers, 2012), ‘positive health’ (e.g. De Jong-Witjes et al. 2022), and ‘appraisal’ (e.g. Gusler et al. 2022). Existing frameworks and theories should be used to guide conceptualization, such as the Sibling Embedded Systems Framework (Kovshoff et al. 2017).

Moreover, the target group, in terms of age and disabilities, should be determined. Some studies include siblings of children with a broad range of conditions (e.g. Blamires et al. 2024) and thus assume that their experiences are quite similar. Others select a more specific sample and argue that the nature of the disability impacts siblings’ experiences (e.g. Shivers et al. 2019). The scale could either focus on a broader target group, or different subgroup versions could be developed. In addition, it should be discussed if self-report and parent-report versions of the scale should measure the same or complementing dimensions (e.g. Dinç et al. 2024). Moreover, the possibility of including a version to capture the perspective of the child with a disability is debatable (e.g. Cebula et al. 2025).

Next, existing sibling-tailored measures (e.g. *Sibling Need and Involvement Profile*; Senner & Fish 2012), or broader measures of adjustment to events or situations (e.g. measures of appraisal; Gusler et al. 2022), should be identified. The usability of these measures as they are, or items of these measures should then be evaluated. From our study, we have learned that although the NAS does not fulfill the requirements, some items are usable. Insights about the experiences and adjustment of siblings from previous qualitative studies (e.g. Múries-Cantán et al. 2023; Schumann et al. 2024) can be used as input for new item generation. Important items within the composed dimensions can be determined in focus groups. Because we aim to develop a scale that can be used internationally, it is of importance that close collaboration between native speakers is in place, to make sure the created items have the same meaning across languages. Next, the content validity of the generated items should be determined, and modifications should be made based on expert and user evaluations, including siblings and parents.

In the second phase, the items and scale are further validated using multiple rounds of cognitive interviews with users. Modifications to phrasing are then made where needed. Next, a larger, diverse group of users will complete all potential items at two measurement points. Basic psychometric properties, such as factor structure and inter-item correlations will be determined. Items that do not function well will be omitted in this phase, aiming for a short questionnaire. Then, in the third phase, the dimensionality, reliability, and validity of the scale will be further evaluated. This should include invariance analysis across age groups, disabilities, and languages.

In summary, in response to our psychometric study into the NAS (Veerman et al. 2024), Supianto (2025) stressed the need to develop a new sibling-tailored measure of adjustment. A rigorous approach, involving an international team of researchers, clinicians, siblings, and parents, is crucial. This process will take multiple years to complete. In the meantime, the NAS appears to be the best available sibling-tailored measure, but it should be used with caution in diverse samples, and be complemented with measures of positive outcomes. We encourage researchers in the field, from across the globe, to reach out to collaborate with us in developing this measure, following the steps outlined in this Reply.

## Data Availability

There is no data to this Reply.
